# Migration Status and Smoking Behaviors in Later-Life in China—Evidence From the China Health and Retirement Longitudinal Study (CHARLS)

**DOI:** 10.3389/fpubh.2018.00346

**Published:** 2018-11-23

**Authors:** Bo Hou, James Nazroo, James Banks, Alan Marshall

**Affiliations:** ^1^National School of Development, Peking University, Beijing, China; ^2^Sociology, School of Social Sciences, The University of Manchester, Manchester, United Kingdom; ^3^Manchester Institute for Collaborative Research on Ageing, School of Social Sciences, The University of Manchester, Manchester, United Kingdom; ^4^Cathie Marsh Institute for Social Research, School of Social Sciences, The University of Manchester, Manchester, United Kingdom; ^5^Economics, School of Social Sciences, The University of Manchester, Manchester, United Kingdom; ^6^The Institute for Fiscal Studies, London, United Kingdom; ^7^School of Social and Political Science, University of Edinburgh, Edinburgh, United Kingdom

**Keywords:** smoking behaviors, smoking cessation, internal migration, return migration, rural-to-urban migration, China

## Abstract

**Background:** China is the biggest consumer of tobacco in the world, with a high prevalence of smoking especially among men. Along with the rapid demographic change in China, the burden of diseases attributable to health behaviors, particularly smoking is steadily increasing. So, smoking has become a major risk factor for mortality in China. Smoking behaviors may be related to migration processes, as a result of both who migrates and post-migration experiences related to socioeconomic position, stress and acculturation. Existing studies that have examined smoking and migration in China have, however, only focused on temporary rural-to-urban migrants and focused on relatively younger migrants. This paper examines the association between smoking behaviors and a comprehensive assessment of migration status in later-life in China.

**Methods:** Using the China Health and Retirement Longitudinal Study (CHARLS), a nationally representative dataset, this paper studies smoking behaviors of rural-to-urban migrants, urban-to-urban migrants, rural return migrants, and urban return migrants. We compare them with corresponding non-migrant groups in both rural and urban locations in China. Using a model that controls for demographic factors, early-life circumstances, socioeconomic factors, and factors related to migration, we examine both the decision to start smoking and the decision to quit smoking. In addition, we also address pre-migration selection in our analyses.

**Results:** The results show rural-to-urban migrants are no more likely to start smoking compared with rural non-migrants, but they are more likely to quit smoking. While urban-to-urban migrants are more likely to start smoking compared with urban non-migrants, this effect is explained by the factors we include in the full model. Urban-to-urban migrants are, however, less likely to quit smoking. Moreover, both rural return migrants and urban return migrants seem to be more likely to start smoking and less likely to quit smoking compared with non-migrant groups.

**Conclusion:** There are strong associations between migration status and later-life smoking behaviors in China; these associations vary greatly according to different migration status and point to populations and factors that public health activities should focus on.

## Introduction

It is estimated that there were over 300 million current smokers in China in 2010, making China the biggest consumer of tobacco in the world (1). Studies on tobacco use in China have found a high prevalence of smoking among men and a low prevalence among women (2, 3). According to a recent survey, 53% of Chinese men, and 2% of the women, and 28% of the total population smoke tobacco products (1). Smoking is a major risk factor for mortality in China (4, 5) and for many chronic diseases, such as stroke (6, 7). Treatment of smoking related diseases in China makes up about 6% of total medical expenditure (8). Therefore, smoking is a particularly important public health issue in China.

The urban population in China grew by 440 million from 1978 to 2011, with rural-to-urban migration accounting for roughly half of this growth, and the other half of this growth due to urban expansion into rural areas (9). This trend is projected to grow further under the current government policy (10). Smoking may relate to migration processes as a result of selection processes occurring before migration and of post-migration circumstances in a more westernized urban environment. Studies have shown that migration is selective in terms of health and levels of education in many countries (11–13). Migration processes may also be selective of particular types of personalities (14), thus it may be related to risky health behaviors, such as smoking (15, 16). In China, rural-to-urban migrants often have better socioeconomic status compared with rural natives and worse socioeconomic status compared with urban natives (17). It is common that these migrants work in jobs such as manufacturing and construction; they often have lower incomes, no social benefits, work very long hours and have very basic living standards (14). Studies have shown that material and cultural disadvantages, and stress, are positively associated with smoking (18–21). As a result of these disadvantages, migrants might have worse smoking behaviors. In addition, features of migration, such as the length of migration are also associated with smoking (19, 20). This might be linked to acculturation after migration.

Few studies have examined smoking cessation in the context of migration. Those that have explored the reasons given by migrants to stop smoking, and have given typical responses such as: prevent future illness, current illness, family pressures and financial considerations (8, 22). Given the range of factors related to smoking among migrants, described above, it is valuable from a public health perspective to also study factors related to smoking cessation.

Also, few studies have looked at smoking behaviors among different types of migrants in China, and those that have provided inconclusive results. Some studies find that rural-to-urban migrants have higher smoking rates (19, 23). While other studies find lower smoking rates among rural-to-urban migrants (16, 20, 22). For instance, using a cross-sectional sample from Beijing, Chen et al. find a similar smoking prevalence for male rural-to-urban migrants compared with their non-migrant counterparts, but a much higher smoking prevalence for female migrants (19). Whereas, Mou et al. report a lower smoking prevalence for migrant workers in Shenzhen compared with national rates and they go on to argue this difference may partly be due to the healthy migrant effect (16).

In addition, return migration is also an integral part of migration processes, many migrants return to the area that they originated from Wang and Fan (24), Zhao et al. (25), and Koser (26). The factors that can influence the migrants' decision to return or move closer to home include: poor health, difficulties in finding jobs, constraints in affording and utilizing health care services in their new locations, and various sources of work-related stress (17, 24, 27). Return migration may also be selective in relation to health, sometimes referred to as salmon bias (28), a hypothesis developed in the US context that states that unhealthy immigrants living in the US tend to return home to die. Return migration may also relate to smoking behaviors (15), because of factors related to the causes of returning.

In this paper, we investigate smoking behaviors and their associations with different types of migration processes in China, examining both the decision to smoke and the decision to stop smoking. In addition, to more thoroughly explore the association with migration, rather than just studying rural-to-urban migrants, we also look at urban-to-urban migrants, rural return migrants and urban return migrants in China. In order to study these processes, we focus on those aged 45 or older, who consequently have more complete histories of migration and of starting and quitting smoking.

## Methods

This paper uses the China Health and Retirement Longitudinal Study (CHARLS), a nationally representative, multi-disciplinary and public dataset that aims to capture the health and well-being of the Chinese population aged 45 and over (29). The CHARLS contains detailed information of respondents' social, economic, and health conditions. Further details on the sample are provided elsewhere (30). This paper uses the CHARLS national baseline survey which was conducted between June 2011 and March 2012. The national baseline survey comprises information on about 17,000 individuals and 10,000 households. Our reasons for choosing the CHARLS baseline survey are: first, the CHARLS sample of older adults contains sufficient people who have been through different types of migration processes, which younger cohorts may not have; second, it includes detailed information on individuals' socioeconomic circumstances and health including early-life circumstances.

Classifying migrants is problematic, this is due to the definition of migrants not being standardized and sometimes unclear; for example, economic migrants can at the same time be illegal migrants and refugees (26). In migration studies in China, a migrant is typically defined as: someone who comes from rural areas and works in an urban area, this person does not have an urban Hukou, and this person is an adult and not a student; for an example, see (31). The Hukou system is a unique feature of migration in China that is loosely similar to an internal passport system, which restricts people's mobility and is linked to access to local welfare and resources (32, 33). There are two types of Hukou, an agricultural type and a non-agricultural type; this classification is based on the rural/urban classification of a person's birthplace. Clearly, these definitions only apply to one type of migration in China, the temporary rural-to-urban migration, i.e., rural-to-urban migrants who have a rural Hukou.

To study wider migration processes in China, this paper classifies migrants according to geographical mobility, with distinctions drawn between movements between and within rural and urban communities in China. This is because of existing inequalities between rural and urban areas in China, and migration is partially driven by inequality in development (26, 34) In particular, we classify the sample into six types: rural non-migrants, urban non-migrants, rural-to-urban migrants, urban-to-urban migrants, rural return migrants, and urban return migrants. The CHARLS uses the classification of an urban area from the National Bureau of Statistics in China, which states a community is urban if it is located in a city, suburb of a city, a town, or other special areas, where non-farming employment constitutes at least 70% of the work force.

The respondents in CHARLS were asked “where were you born?” The answer to this question has five options to choose from, “this village,” “neighborhood in this county or city,” “another county or city in this province,” “another province,” and “abroad.” Using this information, a migrant here is defined as a person whose current place of residence is different from his or her birthplace and not in the surrounding town or city of her birthplace. According to the rural/urban classification of migrants' birthplaces, current places of residences and their Hukou information. Migrants can be further divided into rural-to-urban migrants with a rural Hukou and urban-to-urban migrants. Moreover, for those whose current places of residence are the same or in the neighborhood of their birthplaces, they were asked “Have you ever lived outside this county or city for more than 6 months?” Based on this, return migrants are defined as people who have been outside of their birthplace for more than 6 months, but they were living at their birthplace when they were interviewed. Thus, this definition also includes historical return migrants. Unfortunately, however, the data is not detailed enough to be able to build in information about how many times they have left for more than 6 months. Non-migrants are defined as people whose current places of residence are the same or in the neighborhood of their birthplaces, and they have never had any migration experience that is longer than 6 months. In addition, using information on the timing of the initial migration, we exclude migrants and return migrants who migrated in their childhood, because our focus is on adult migration effects.

We use the question “have you ever chewed tobacco, smoked a pipe, smoked self-rolled cigarettes, or smoked cigarettes/cigars?” to identify those who have ever smoked. This question further defines smoking as having smoked at least 100 cigarettes or equivalent in a respondent's lifetime. To cover the decision to quit smoking the questionnaire includes the question “do you still have the habit or have you totally quit?” These behaviors are modeled in two stages: in the first stage, we examine predictors of starting smoking for everyone; the second stage examines predictors of quitting smoking for those who have ever smoked. These outcome variables are binary and were modeled using logistic regressions.

In the empirical model, we control for factors related to demography, early-life selection, current socioeconomic circumstances, and migration. Demographic factors include age, gender and marital status. Early-life factors include lower leg length (knee height), education and first job. The relationship between educational attainments and smoking is debated. Generally, there is a negative relationship between smoking rates and levels of education; but evidence also shows that smoking rates for people with relatively high levels of education could also be high (2, 8) This might be because smoking symbolizes greater social status in China (16). As migrants' smoking behaviors may relate to the healthy migrant effect, we also account for factors related to the selective features of migration. We deal with the selection of migrants and the selection of returnee's initial migration by controlling for pre-migration markers of health and economic selection, e.g., lower leg length, a proxy for youth health, and childhood socioeconomic circumstances (35). For socioeconomic factors, this model includes measures of current job status, annualized expenditure, households' consumer durables and house ownership. Factors related to migration processes are the time since migration to the place of destination and participation in local social activities post-migration. Participation in local social activity is an indicator for levels of acculturation at the place of destination as it may capture some level of social integration and the presence of a social network (36). For models of stopping smoking, we also additionally include current self-reported health status, as poor health may be a predictor of quitting smoking (8).

This paper uses a progression of regressions to analyse the relationship between smoking behaviors and migration status in China, adding in each cluster of factors sequentially into the empirical model. As there are many different migrant statuses, and there is an existing inequality between the rural and urban China (34), our models are stratified according to rural and urban for clarity. Effectively, the different migrant groups will be operated as two treatment variables: one includes rural groups (rural non-migrants, rural-to-urban migrants, and rural return migrants) and the other includes urban groups (urban non-migrants, urban-to-urban migrants and urban return migrants). To provide information on differences in smoking behaviors between rural and urban non-migrants, we also run the same models on these two groups. These results are presented in the Supplementary Material. To account for potential heteroscedasticity, robust standard errors are used in all regressions. Sampling weights were incorporated into these analyses. Analyses were conducted using STATA 14 (StataCorp, College Station, TX, USA). Full results are in the Supplementary Material. In addition, we have truncated the sample at 80; about 3% of the CHARLS sample is over this age.

## Results

### Selection

Table 1 shows the distribution of variables in this empirical model stratified by migration status. This table shows that the majority of return migrants are male, i.e., 75%. There are slightly more females in both migrant groups compared with the non-migrant groups. In terms of the objective measure for youth nutrition, return migrants have higher lower leg length compared with both the migrant and non-migrant groups. Migrants to urban areas have higher lower leg length compared with the urban non-migrant group. For instance, the mean of lower leg length for urban return migrants is 48.85 cm, while it is 48.68 cm for urban-to-urban migrants and is 48.33 cm for urban non-migrants. This figure for rural-to-urban migrants is 47.72, 47.75 cm for rural non-migrants and is 48.93 cm for rural return migrants. Moreover, return migrants seem to have better education than non-migrants, but they have worse education compared with migrants, except for rural-to-urban migrants with a rural Hukou. For instance, 17.6% of rural return migrants have no formal education, which compares with 36.45% of rural non-migrants, and 24.5% of rural-to-urban migrants.

**Table 1:** Variable distribution and migration status.

**Variables list**	**Rural non-migrants**	**Rural-to-urban**	**Rural Return migrants**	**Urban non-migrants**	**Urban to urban**	**Urban return migrants**	**Sample Sizes**
Decision to smoke (%)	40%	33%	63%	34%	28%	58%	12,736
Decision to stop smoking (%)	19%	28%	18%	24%	33%	23%	5,301
Age	58.56	56.76	57.29	57.11	62.75	58.15	12,806
Male (%)	47%	34%	78%	46%	40%	75%	12,818
**MARITAL STATUS (%)**							
Married with spouse present	80%	77%	71%	84%	79%	80%	10,212
Married not living with spouse temporarily	6%	10%	18%	4%	2%	11%	937
Separated, divorced, widowed and	14%	13%	11%	12%	19%	9%	1,673
**NEVER MARRIED**							
Knee height	47.75	47.72	48.93	48.33	48.69	48.85	9,847
**EDUCATION (%)**							
No formal education	36%	25%	18%	10%	7%	7%	3,558
Primary education	41%	45%	47%	27%	21%	35%	4,937
Secondary education	22%	29%	34%	49%	44%	41%	3,696
Tertiary education	1%	2%	2%	14%	28%	17%	615
**FIRST JOB (%)**							
Government	1%	1%	2%	5%	9%	8%	240
Institutions	2%	2%	3%	15%	23%	11%	595
State firms	1%	3%	5%	42%	48%	25%	1,192
Individual firms	1%	6%	2%	4%	2%	3%	245
Farmers	92%	85%	82%	28%	11%	44%	8,790
Other occupations	2%	3%	5%	5%	7%	8%	432
**CURRENT JOB STATUS**							
Agricultural work	71%	27%	65%	8%	1%	28%	7,027
Wage work	6%	29%	16%	35%	19%	30%	1,732
Retired and receive a public pension	5%	6%	4%	27%	50%	20%	1,242
Retired and receive no pension	12%	20%	6%	13%	15%	9%	1,449
Not working	7%	18%	8%	18%	16%	13%	1,209
Annualized expenditure on food	7.38	10.98	7.78	12.01	15.41	11.53	12,084
Annualized expenditure on other things	13.27	24.52	15.33	21.8	28.97	23.52	12,623
Household durables wealth	6.47	10.74	6.41	11.46	22.74	11.97	12,811
**HOUSE OWNERSHIP**							
None	8%	33%	9%	12%	17%	10%	1,202
Partially	5%	2%	7%	2%	7%	3%	584
Fully	87%	65%	85%	85%	77%	87%	10,837
Length of migration	0	22.84	6.31	0	31.65	8.02	12,729
**SOCIAL ACTIVITY**							
None	55%	57%	48%	43%	37%	39%	5,811
One type	35%	32%	33%	35%	36%	37%	3,925
Two types	10%	9%	18%	17%	18%	19%	1,365
Three types	0%	1%	1%	5%	9%	5%	190
**HEALTH STATUS**							
Excellent and very good	6%	12%	8%	9%	11%	9%	940
Good	15%	17%	15%	19%	20%	18%	2,030
Fair	44%	43%	47%	51%	53%	52%	5,861
Poor	31%	25%	28%	19%	15%	20%	3,567
Very poor	3%	2%	2%	2%	1%	2%	353
Sample sizes for migrant groups	8,220	349	1,234	2,052	285	685	12,825

### Current socioeconomic circumstances

Table 1 also shows that migrants have much higher values of household durable wealth compared with the other two groups. Return migrants have similar household durable wealth compared with non-migrant groups. For example, the average of household durables wealth is 11,460 yuan for urban non-migrants, 11,970 yuan for urban return migrants and 22,740 yuan for urban-to-urban migrants. For annualized spending on things other than food, return migrants have higher spending compared with non-migrant groups, but lower spending compared with migrant groups. The annualized non-food expenditure for rural non-migrants is 13,270 yuan, 15,330 yuan for rural return migrants, and 24,520 yuan for rural-to-urban migrants.

### Features related to migration

For the variables related to features of migration, the average length of migration is 22.84 years for rural-to-urban migrants and 6.31 years for rural return migrants. For urban groups it is 31.65 years for urban-to-urban migrants and 8.02 years for urban return migrants. For current social activities, return migrants seem to be more socially active compared with their non-migrant and migrant counterparts.

### Self-rated health status

Migrants report the best scores of self-reported health status. Rural return migrants have slightly better self-reported health compared with rural non-migrants. But urban return migrants have slightly worse self-reported health status compared with urban non-migrants. For instance, 27.24% of rural-to-urban migrants reported poor and very poor health status, compared with 29.63% of rural return migrants and 34.66% of rural non-migrants. In the urban area, 21.31% of urban non-migrants reported poor and very poor health status, compared with 16.43% of urban-to-urban migrants and 21.91% of urban return migrants.

### Association between smoking behaviors and migration status

From Table 1, 40% of rural non-migrants have smoked, while 34% of urban migrants have smoked. compared with migrants, return migrants have the greatest proportion of people that have ever smoked and migrants have the lowest proportion of people who have ever smoked. For instance, 63% of rural return migrants have smoked, compared with 40% of rural non-migrants, and 33% of rural-to-urban migrants. In urban areas, 58% of urban return migrants have ever smoked before, the figures for urban-to-urban migrants and urban non-migrants are 28 and 34%, respectively. In terms of quitting smoking, compared with non-migrant groups, migrant groups have the highest proportion of people that have stopped smoking and return migrants have the lowest proportion of people that have stopped smoking. In rural areas, 28% of rural-to-urban migrants who have ever smoked have stopped, compared with 19% of rural non-migrants, and 18% rural return migrants. In urban areas, 33% of ever smokers in urban to urban migrant group have stopped smoking, compared with 24% of urban non-migrants and 23% of urban return migrants.

Table 2 presents models for ever smoking for those from rural areas. This shows that there are no statistically significant differences between rural-to-urban migrants and rural non-migrants across all models. Rural return migrants seem to be more likely to start smoking compared with rural non-migrants. Although this difference is explained after adjusting for sex, it becomes statistically significant again after adjusting for differences in current socioeconomic factors. But this difference is explained in the final model after adjusting for factors related to migration. The odds ratio for rural return migrants is 2.654 (*p* < 0.001) after adjusting for age. It reduces dramatically and becomes 1.207 (*p* > 0.05) after controlling for sex. This is because the majority of returnees are men and smoking is more common among men (almost 90% of smokers are men). This odds ratio changes slightly to 1.22 (*p* > 0.05) after adjusting for early-life selective factors. But after adjusting for current socioeconomic factors this odds ratio becomes 1.246 (*p* < 0.05) in model 4. In the final model which additionally accounts for factors related to migration and levels of social activity, the odds ratio for this group is 1.098 (*p* > 0.05).

**Table 2:** Decision to smoke logistic regressions (rural base).

**Model specifications**	**1**	**2**	**3**	**4**	**5**
	**+Age**	**+Sex**	**+Early-life**	**+Socioeconomic**	**Full**
Rural non-migrants					
Rural-to-urban migrants	0.796	1.048	1.052	1.181	0.869
	(0.412–1.538)	(0.456–2.410)	(0.477–2.320)	(0.620–2.250)	(0.361–2.091)
Rural return migrants	2.654^***^	1.207	1.220	1.246^*^	1.098
	(2.249–3.132)	(0.981–1.485)	(0.986–1.510)	(1.005–1.545)	(0.856–1.409)
*N*	6,935	6,935	6,935	6,935	6,935
Pseudo R-sq	0.0224	0.3938	0.3968	0.3999	0.4006

Odds ratios in the table

* *p < 0.05*,

** *p < 0.01*,

*** *p < 0.001. Ninety-five percent confidence intervals in parentheses, calculated based on robust standard errors*.

Findings for the urban groups are shown in Table 3. Urban return migrants are more likely to start smoking compared with urban non-migrants. The odds ratio for this group is 1.699 (*p* < 0.01) after adjusting for age and sex in model 2. Then it largely stays around this level in later models. This odds ratio is 1.7 (*p* < 0.01) after adjusting for early-life factors, is 1.714 (*p* < 0.01) after controlling for socioeconomic factors and is 1.79 (*p* < 0.01) after adjusting for factors related to migration in the final model. For urban-to-urban migrants, there is a statistically significant increased risk of ever smoking after adjusting for age and sex. Adjusting for early-life and socioeconomic factors attenuates this relationship slightly. But this difference is explained after adjusting for factors related to migration in the final model. For instance, the odds ratio for this group is 1.479 (*p* > 0.05) after adjusting for age. It becomes 1.875 (*p* < 0.05) after adjusting for differences in sex. It is 2.216 (*p* < 0.05) after adjusting for early-life factors in model 3 and is 1.927 (*p* < 0.05) after controlling for differences in socioeconomic factors. Finally, it is 2.366 (*p* > 0.05) after controlling for factors related to migration in the final model. In addition, from Table S5, the results show that urban non-migrants are less likely to start smoking compared with rural non-migrants. For instance, the odds ratio for urban non-migrants is 0.597 (*p* < 0.001) after controlling for age and sex. It becomes 0.701 (*p* < 0.05) after controlling for early-life and socioeconomic factors and is 0.697 (*p* < 0.05) in the full model.

**Table 3:** Decision to smoke logistic regressions (urban base).

**Model specifications**	**1**	**2**	**3**	**4**	**5**
	**+Age**	**+Sex**	**+Early-life**	**+Socioeconomic**	**Full**
Urban non-migrants					
Urban-to-urban migrants	1.479	1.875^*^	2.216^*^	1.927^*^	2.366
	(0.685–3.191)	(1.007–3.489)	(1.199–4.096)	(1.117–3.325)	(0.956–5.851)
Urban return migrants	3.401^***^	1.699^**^	1.700^**^	1.714^**^	1.790^**^
	(2.594–4.459)	(1.217–2.373)	(1.202–2.404)	(1.203–2.442)	(1.202–2.666)
*N*	1,713	1,713	1,713	1,713	1,713
pseudo R-sq	0.0555	0.3588	0.3728	0.3814	0.3958

Odds ratios in the table

* *p < 0.05*,

** *p < 0.01*,

*** *p < 0.001. Ninety-five percent confidence intervals in parentheses, calculated based on robust standard errors*.

Table 4 shows that rural-to-urban migrants are more likely to quit smoking compared with the rural non-migrants. The odds ratio for this group is 6.481 (*p* < 0.001) after controlling for age and sex in model two. Adjusting for early-life factors slightly attenuates this to 5.901 (*p* < 0.001). This odds ratio becomes 3.832 (*p* < 0.01) after controlling for current socioeconomic factors and it is 7.1 (*p* < 0.001) after additionally adjusting for factors related to migration and current health status. In terms of rural return migrants, the results show that there are no statistically significant differences between rural return migrants and rural non-migrants.

**Table 4:** Decision to stop smoking logistic regressions (rural base).

**Model specifications**	**1**	**2**	**3**	**4**	**5**
	**+Age**	**+Sex**	**+Early life**	**+Socioeconomic**	**Full**
Rural non-migrants					
Rural-to-urban migrants	6.486^***^	6.481^***^	5.901^***^	3.832^**^	7.100^***^
	(2.461–17.092)	(2.449–17.152)	(2.264–15.379)	(1.682–8.727)	(2.464–20.459)
Rural return migrants	0.962	0.963	0.979	1.007	1.257
	(0.744–1.243)	(0.744–1.247)	(0.753–1.272)	(0.772–1.314)	(0.932–1.696)
*N*	2,963	2,963	2,963	2,963	2,963
pseudo R-sq	0.037	0.037	0.0449	0.0609	0.0766

Odds ratios in the table ^*^p < 0.05,

** *p < 0.01*,

*** *p < 0.001. Ninety-five percent confidence intervals in parentheses, calculated based on robust standard errors*.

Table 5 shows the results for quitting smoking for urban areas. Compared with urban non-migrants, urban-to-urban migrants are less likely to stop smoking. The odds ratio for this group is 0.379 (p < 0.05) after adjust for age and sex in model two. It is 0.341 (p < 0.05) after additionally adjusting for early-life factors, is 0.361 (p < 0.05) after controlling for current socio-economic factors, and is 0.314 (p < 0.05) in the final model after accounting for factors related to migration and current health status. Similar to the results of rural return migrants in Table 4, there are no statistically significant differences in decisions to quit smoking between urban return migrants and urban non-migrants. Additionally, from Table S6, the results show that urban non-migrants are more likely to stop smoking compared with rural non-migrants after adjusting for differences in age and sex. But this difference is explained by controlling for early-life factors, such as youth nutrition. For instance, the odds ratio for urban non-migrants is 1.452 (p < 0.05) after controlling for age and sex. It becomes 1.293 (p > 0.05) after controlling for early-life factors and is 1.101 (p > 0.05) in the full model.

**Table 5:** Decision to stop smoking logistic regressions (urban base).

**Model specifications**	**1**	**2**	**3**	**4**	**5**
	**+Age**	**+Sex**	**+Early life**	**+Socioeconomic**	**Full**
Urban non-migrants					
Urban-to-urban migrants	0.379^*^	0.379^*^	0.341^*^	0.361^*^	0.314^*^
	(0.161–0.893)	(0.161–0.891)	(0.139–0.837)	(0.150–0.868)	(0.103–0.957)
Urban return migrants	0.877	0.888	0.916	0.894	0.889
	(0.571–1.348)	(0.574–1.372)	(0.597–1.407)	(0.569–1.405)	(0.528–1.497)
N	676	676	676	676	676
pseudo R-sq	0.0421	0.0423	0.0671	0.0965	0.125

Odds ratios in the table

* *p < 0.05*,

^**^ p < 0.01, ^***^ p < 0.001.

*Ninety-five percent confidence intervals in parentheses, calculated based on robust standard errors*.

For the control variables in tables of starting to smoke, social activities, a measure of levels of social integration, shows a statistically significant and positive relationship with the decision to start smoking in urban areas. But this does not hold in the rural sample. Forty-seven percent of smokers in the rural sample participated in at least one type of social activity, this compares to 62% of smokers in the urban sample. This may suggest that social activities in urban arears have a stronger association with smoking initiation (37). Compared with having no formal education, having a tertiary education shows statistically significant and negative relationship with the decision to start smoking, but having a primary or a secondary education shows no significant relationship. That rural-to-urban migrants are more likely to quit smoking may be related to financial considerations. Compared with rural non-migrants in Table 1, almost double the proportion of rural-to-urban migrants are retired and receive no pension and are in non-working groups. This may also be related to their agricultural hukou status, which limits their access to local welfare and resources. The results from Table S3 shows smoking cessation is strongly associated with being in these two groups compared with doing agriculture work. For the control variables in tables of quitting smoking, length of migration shows a negative relationship with the decision to stop smoking in rural sample, with an odds ratio of 0.967 (*p* < 0.05). But this is not present in urban sample. Moreover, there is a statistically significant and negative relationship between the decision to stop smoking and current self-reported health status. This is true for both rural and urban samples. This may partly explain why urban-to-urban migrants are less likely to quit smoking as they have better self-reported health, for instance 15% of urban migrants reported poor health compared to 19% of urban non-migrants. These results are shown in full in the Supplementary Material.

## Discussion

Previous studies on smoking behaviors and migration in China have largely looked at one type of migration, rural-to-urban migration; and the results are inconclusive. This may partly be due to heterogeneity among migrants (38). This paper investigates the relationship between smoking behaviors and a fuller range of types of migration in China, covering rural to urban migration, but also migration within rural and urban areas and including return migration. We have also examined both the decision to smoke and the decision to quit smoking. To do this, we use a nationally representative dataset, the CHARLS, and an empirical model informed by the literature. This paper also addresses the selective features of migration, by excluding early-life migrants and adding measures of early-life nutrition and socioeconomic position into the analyses.

Our results show that there is a strong association between smoking behaviors and migration status in later-life in China. Compared with non-migrants, migrants who have returned to their place of origin have the greatest proportion of people who have ever smoked and have the lowest proportion of ever smokers who have stopped smoking. Migrants who have not returned have the lowest proportion of people who have ever smoked and have the highest proportion of people who have stopped smoking, except for urban-to-urban migrants. We also find smoking is more prevalent in rural areas in China compared to urban areas. Urban non-migrants are less likely to start smoking compared to rural non-migrants, and they are more likely to stop smoking partly due to early-life factors. Different types of migration processes are associated with smoking behaviors differently. First, there are no statistically significant differences between rural-to-urban migrants and rural non-migrants to have ever smoked across all our models. However, rural-to-urban migrants are more likely to quit smoking compared with the rural non-migrants. Second, urban-to-urban migrants are more likely to smoke than urban non-migrants. But this difference is explained after adjusting for factors related to migration, particularly social activities, a measure reflecting levels of social integration. Moreover, urban-to-urban migrants are less likely to stop smoking compared with urban non-migrants. Third, rural return migrants seem to be more likely to start smoking compared with rural non-migrants. Although this difference is explained after adjusting for gender, it becomes statistically significant again after adjusting for differences in current socioeconomic factors. But this difference is explained in the final model after adjusting for factors related to migration. In urban areas, urban return migrants are more likely to start smoking compared with urban non-migrants. Furthermore, there are no statistically significant differences in terms of the decision to quit smoking between return migrants from both rural and urban locations and non-migrants in rural and urban areas, respectively. Note that these return migrants are people who had a migration experience, thus this may suggest that return migration may have lasting effects on smoking behaviors after migration in adult life, as having experienced an episode of migration is negatively associated with smoking behaviors. The exact mechanism may be associated with reasons for returning, such as poor health (15). In addition, our results also find that poorer current health strongly predicts quitting smoking.

There are important limitations to this study. The data are collected cross-sectionally and causal inferences cannot be straightforwardly drawn from these results, although we have controlled for some self-selective features of migration. In addition, because of limitations in the coverage of the data, the timing of initial migration for returnees may contain some errors, thus we may have overestimated the length of migration for return migrants.

These findings contribute to the literature by examining the relationship between smoking behaviors and a fuller range of migration status in China. Rather than looking at migrants and non-migrants, we have separately examined migrants according to their place of origin, and have also identified those migrants who have returned to their places of origin. As return migrants seem to have worse smoking behaviors than non-migrants, neglecting this group of people may cause bias in attempts to explore the association between migration and smoking behaviors. A fruitful topic for future research is to look into the mechanisms through which return migration affects smoking behaviors. Also, our findings contribute to the literature by looking at the older population in China. Rapid demographic and epidemiological changes in China have taken place over the last several decades, the burden of diseases related to health behaviors is steadily increasing and smoking has become a major risk factor for ill-health and disability (39). The benefits of smoking cessation are significant and often underestimated, stopping smoking even at middle age can substantially reduce the risk of smoking-related death (40). Strengthening government policies for smoking prevention and cessation is required in China to decrease smoking-related morbidity and mortality. Given the size of internal migration and its projected future trend in China, national policies that target the returned migrant population may be particularly effective in terms of smoking prevention and cessation.

## Author contributions

BH conducted the analyses and wrote the manuscript. JN, JB, and AM guided the data analysis procedures and reviewed the manuscript. All authors approved the final version of the manuscript.

### Conflict of interest statement

The authors declare that the research was conducted in the absence of any commercial or financial relationships that could be construed as a potential conflict of interest.
