# Supplementary material for: Migration Status and Smoking Behaviors in Later-Life in China—Evidence From the China Health and Retirement Longitudinal Study (CHARLS)
Source: Front Public Health. 2018 Nov 23;6:346. doi: 10.3389/fpubh.2018.00346 (PMC6266545; doi:10.3389/fpubh.2018.00346)
Supplement: Supplementary file 1 [file Table_1.DOCX]

S1 Decision to Smoke Logistic Regressions (Rural base)

|  | 1 | 2 | 3 | 4 | 5 |
| --- | --- | --- | --- | --- | --- |
| Model specifications | +Age | +Sex | +Early life | +Socioeconomic | Full |
| Rural non-migrants | . | . | . | . | . |
| Rural-to-urban | -0.228 | 0.047 | 0.051 | 0.167 | -0.140 |
|  | (0.336) | (0.425) | (0.404) | (0.329) | (0.448) |
| Rural return migrants | 0.976*** | 0.188 | 0.199 | 0.220* | 0.093 |
|  | (0.084) | (0.106) | (0.109) | (0.110) | (0.127) |
| Centered-Age | 0.021*** | 0.017*** | 0.012* | 0.008 | 0.008 |
|  | (0.004) | (0.005) | (0.005) | (0.006) | (0.006) |
| Centered-Age squared | -0.001** | -0.001 | -0.001 | -0.001 | -0.001* |
|  | (0.000) | (0.000) | (0.000) | (0.000) | (0.000) |
| Female as base |  | . | . | . | . |
| Male |  | 3.567*** | 3.559*** | 3.611*** | 3.622*** |
|  |  | (0.086) | (0.104) | (0.104) | (0.104) |
| Married with spouse present |  |  | . | . | . |
| Married not living with spouse temporarily |  |  | 0.041 | 0.039 | 0.068 |
|  |  |  | (0.215) | (0.211) | (0.220) |
| Separated, divorced, widowed and never married |  |  | 0.254 | 0.251 | 0.254 |
|  |  |  | (0.172) | (0.163) | (0.163) |
| knee height |  |  | 0.028* | 0.028* | 0.027* |
|  |  |  | (0.013) | (0.013) | (0.013) |
| No formal education |  |  | . | . | . |
| Primary |  |  | -0.088 | -0.098 | -0.102 |
|  |  |  | (0.098) | (0.098) | (0.099) |
| Secondary |  |  | -0.184 | -0.198 | -0.204 |
|  |  |  | (0.126) | (0.122) | (0.121) |
| Tertiary |  |  | -0.669* | -0.707* | -0.723* |
|  |  |  | (0.279) | (0.286) | (0.282) |
| First job as farmers base |  |  | . | . | . |
| First job in the Government |  |  | 0.137 | 0.149 | 0.105 |
|  |  |  | (0.401) | (0.410) | (0.415) |
| First job in Institutions |  |  | -0.156 | -0.156 | -0.159 |
|  |  |  | (0.223) | (0.221) | (0.222) |
| First job in State firms |  |  | 0.202 | 0.232 | 0.209 |
|  |  |  | (0.262) | (0.266) | (0.266) |
| First job in Individual Firms |  |  | -0.626* | -0.563* | -0.586* |
|  |  |  | (0.272) | (0.279) | (0.284) |
| First job in other jobs |  |  | -0.029 | 0.026 | 0.013 |
|  |  |  | (0.466) | (0.415) | (0.417) |
| Current job status, agricultural work |  |  |  | . | . |
| Wage work |  |  |  | -0.149 | -0.136 |
|  |  |  |  | (0.171) | (0.172) |
| Retired and receive any pension |  |  |  | 0.297 | 0.255 |
|  |  |  |  | (0.208) | (0.206) |
| Retired and receive no pension |  |  |  | 0.143 | 0.143 |
|  |  |  |  | (0.145) | (0.145) |
| Not working |  |  |  | -0.586** | -0.572* |
|  |  |  |  | (0.225) | (0.228) |
| Annualised food expenditure |  |  |  | -0.008 | -0.008 |
|  |  |  |  | (0.006) | (0.006) |
| Annualised food expenditure squared |  |  |  | 0.000 | 0.000 |
|  |  |  |  | (0.000) | (0.000) |
| Annualised other expenditure |  |  |  | 0.004 | 0.004 |
|  |  |  |  | (0.004) | (0.004) |
| Annualised other expenditure squared |  |  |  | -0.000 | -0.000 |
|  |  |  |  | (0.000) | (0.000) |
| Household durables |  |  |  | -0.001 | -0.001 |
| continues |  |  |  |  |  |
|  |  |  |  | (0.001) | (0.001) |
| House ownership, none |  |  |  | . | . |
| Partially own |  |  |  | -0.333 | -0.383 |
|  |  |  |  | (0.217) | (0.214) |
| Totally own |  |  |  | 0.029 | -0.014 |
|  |  |  |  | (0.145) | (0.141) |
| Length of migration |  |  |  |  | 0.018 |
|  |  |  |  |  | (0.011) |
| Social activity participation, none |  |  |  |  | . |
| One type |  |  |  |  | 0.019 |
|  |  |  |  |  | (0.082) |
| Two types |  |  |  |  | 0.149 |
|  |  |  |  |  | (0.153) |
| Three types |  |  |  |  | 0.044 |
|  |  |  |  |  | (0.399) |
| Cons | -0.327*** | -2.353*** | -3.639*** | -3.648*** | -3.597*** |
|  | (0.037) | (0.074) | (0.584) | (0.605) | (0.608) |
| N | 6935 | 6935 | 6935 | 6935 | 6935 |
| pseudo R-sq | 0.0224 | 0.3938 | 0.3968 | 0.3999 | 0.4006 |

Robust Standard errors in parentheses * p<0.05 **p<0.01 *** p<0.001

Coefficients are in terms of log odds

S2 Decision to Smoke Logistic Regressions (Urban base)

|  | 1 | 2 | 3 | 4 | 5 |
| --- | --- | --- | --- | --- | --- |
| Model specifications | +Age | +Sex | +Early life | +Socioeconomic | Full |
| Urban non-migrants | . | . | . | . | . |
| Urban-to-urban | 0.391 | 0.628* | 0.796* | 0.656* | 0.861 |
|  | (0.392) | (0.317) | (0.313) | (0.278) | (0.462) |
| Urban return migrants | 1.224*** | 0.530** | 0.531** | 0.539** | 0.582** |
|  | (0.138) | (0.170) | (0.177) | (0.181) | (0.203) |
| Centered-Age | 0.021** | 0.017 | 0.012 | 0.018 | 0.022 |
|  | (0.008) | (0.009) | (0.009) | (0.011) | (0.012) |
| Centered-Age squared | -0.002** | -0.003** | -0.003** | -0.003** | -0.003** |
|  | (0.001) | (0.001) | (0.001) | (0.001) | (0.001) |
| Female as base |  | . | . | . | . |
| Male |  | 3.416*** | 3.554*** | 3.482*** | 3.616*** |
|  |  | (0.189) | (0.219) | (0.219) | (0.223) |
| Married with spouse present |  |  | . | . | . |
| Married not living with spouse |  |  | 0.343 | 0.351 | 0.334 |
| temporarily |  |  | (0.424) | (0.425) | (0.453) |
| Separated, divorced, widowed and |  |  | 0.534 | 0.429 | 0.443 |
| never married |  |  | (0.308) | (0.297) | (0.291) |
| knee height |  |  | 0.018 | 0.016 | 0.012 |
|  |  |  | (0.023) | (0.024) | (0.025) |
| No formal education |  |  | . | . | . |
| Primary |  |  | -0.088 | -0.083 | -0.182 |
|  |  |  | (0.359) | (0.356) | (0.355) |
| Secondary |  |  | -0.304 | -0.291 | -0.422 |
|  |  |  | (0.343) | (0.339) | (0.343) |
| Tertiary |  |  | -1.080** | -1.063** | -1.317*** |
|  |  |  | (0.390) | (0.384) | (0.392) |
| First job as farmers base |  |  | . | . | . |
| First job in the Government |  |  | -0.370 | -0.344 | -0.269 |
|  |  |  | (0.311) | (0.322) | (0.327) |
| First job in Institutions |  |  | 0.335 | 0.413 | 0.464 |
|  |  |  | (0.265) | (0.274) | (0.278) |
| First job in State firms |  |  | 0.027 | 0.015 | 0.054 |
|  |  |  | (0.198) | (0.207) | (0.208) |
| First job in Individual Firms |  |  | -0.029 | -0.007 | -0.037 |
|  |  |  | (0.388) | (0.404) | (0.415) |
| First job in other jobs |  |  | -0.248 | -0.232 | -0.138 |
|  |  |  | (0.350) | (0.357) | (0.363) |
| Current job status, agricultural work |  |  |  | . | . |
| Wage work |  |  |  | -0.138 | -0.212 |
|  |  |  |  | (0.230) | (0.235) |
| Retired and receive any pension |  |  |  | -0.179 | -0.304 |
|  |  |  |  | (0.262) | (0.267) |
| Retired and receive no pension |  |  |  | -0.351 | -0.462 |
|  |  |  |  | (0.349) | (0.349) |
| Not working |  |  |  | 0.544 | 0.436 |
|  |  |  |  | (0.303) | (0.306) |
| Annualised food expenditure |  |  |  | 0.002 | -0.003 |
|  |  |  |  | (0.014) | (0.014) |
| Annualised food expenditure squared |  |  |  | 0.000 | 0.000 |
|  |  |  |  | (0.000) | (0.000) |
| Annualised other expenditure |  |  |  | -0.005 | -0.005 |
|  |  |  |  | (0.004) | (0.004) |
| Annualised other expenditure squared |  |  |  | 0.000 | 0.000 |
|  |  |  |  | (0.000) | (0.000) |
| Household durables |  |  |  | 0.004 | 0.004 |
|  |  |  |  | (0.004) | (0.004) |
| continues |  |  |  |  |  |
| House ownership, none |  |  |  | . | . |
| Partially own |  |  |  | 0.238 | 0.221 |
|  |  |  |  | (0.361) | (0.361) |
| Totally own |  |  |  | -0.241 | -0.308 |
|  |  |  |  | (0.231) | (0.234) |
| Length of migration |  |  |  |  | -0.011 |
|  |  |  |  |  | (0.013) |
| Social activity participation, none |  |  |  |  | . |
| One type |  |  |  |  | 0.625*** |
|  |  |  |  |  | (0.183) |
| Two types |  |  |  |  | 1.002*** |
|  |  |  |  |  | (0.208) |
| Three types |  |  |  |  | 0.765* |
|  |  |  |  |  | (0.330) |
| Cons | -0.580*** | -2.611*** | -3.352** | -2.952* | -2.973* |
|  | (0.086) | (0.177) | (1.104) | (1.180) | (1.221) |
| N | 1713 | 1713 | 1713 | 1713 | 1713 |
| pseudo R-sq | 0.0555 | 0.3588 | 0.3728 | 0.3814 | 0.3958 |

Robust Standard errors in parentheses * p<0.05 **p<0.01 *** p<0.001

Coefficients are in terms of log odds

S3 Decision to Stop Smoking Logistic Regressions (Rural base)

|  | 1 | 2 | 3 | 4 | 5 |
| --- | --- | --- | --- | --- | --- |
| Model specifications | +Age | +Sex | +Early life | +Socioeconomic | Full |
| Rural non-migrants | . | . | . | . | . |
| Rural-to-urban | 1.870*** | 1.869*** | 1.775*** | 1.343** | 1.960*** |
|  | (0.494) | (0.497) | (0.489) | (0.420) | (0.540) |
| Rural return migrants | -0.039 | -0.038 | -0.021 | 0.007 | 0.229 |
|  | (0.131) | (0.132) | (0.134) | (0.136) | (0.153) |
| Centered-Age | 0.026*** | 0.026*** | 0.029*** | 0.022** | 0.020* |
|  | (0.007) | (0.007) | (0.008) | (0.008) | (0.008) |
| Centered-Age squared | 0.000 | 0.000 | 0.000 | -0.000 | 0.000 |
|  | (0.001) | (0.001) | (0.001) | (0.001) | (0.001) |
| Female as base |  | . | . | . | . |
| Male |  | -0.020 | -0.184 | -0.158 | -0.168 |
|  |  | (0.176) | (0.189) | (0.194) | (0.189) |
| Married with spouse present |  |  | . | . | . |
| Married not living with spouse |  |  | 0.518 | 0.474 | 0.419 |
| temporarily |  |  | (0.312) | (0.330) | (0.309) |
| Separated, divorced, widowed and |  |  | -0.151 | -0.213 | -0.231 |
| never married |  |  | (0.168) | (0.175) | (0.172) |
| knee height |  |  | 0.038* | 0.039* | 0.042* |
|  |  |  | (0.019) | (0.019) | (0.019) |
| No formal education |  |  | . | . | . |
| Primary |  |  | 0.093 | 0.098 | 0.113 |
|  |  |  | (0.143) | (0.145) | (0.144) |
| Secondary |  |  | 0.096 | 0.051 | 0.074 |
|  |  |  | (0.170) | (0.172) | (0.171) |
| Tertiary |  |  | 0.306 | 0.337 | 0.411 |
|  |  |  | (0.406) | (0.390) | (0.393) |
| First job as farmers base |  |  | . | . | . |
| First job in the Government |  |  | -0.584 | -0.754 | -0.638 |
|  |  |  | (0.497) | (0.510) | (0.474) |
| First job in Institutions |  |  | 0.005 | -0.101 | 0.003 |
|  |  |  | (0.333) | (0.348) | (0.346) |
| First job in State firms |  |  | -0.056 | -0.180 | -0.062 |
|  |  |  | (0.279) | (0.282) | (0.278) |
| First job in Individual Firms |  |  | -0.194 | -0.252 | -0.097 |
|  |  |  | (0.477) | (0.458) | (0.451) |
| First job in other jobs |  |  | -0.897** | -0.968** | -0.885** |
|  |  |  | (0.327) | (0.330) | (0.333) |
| Current job status, agricultural work |  |  |  | . | . |
| Wage work |  |  |  | 0.221 | 0.249 |
|  |  |  |  | (0.254) | (0.246) |
| Retired and receive any pension |  |  |  | 0.692** | 0.678** |
|  |  |  |  | (0.252) | (0.242) |
| Retired and receive no pension |  |  |  | 0.514** | 0.411* |
|  |  |  |  | (0.190) | (0.185) |
| Not working |  |  |  | 0.689** | 0.526* |
|  |  |  |  | (0.265) | (0.259) |
| Annualised food expenditure |  |  |  | 0.000 | 0.001 |
|  |  |  |  | (0.016) | (0.015) |
| Annualised food expenditure |  |  |  | -0.000 | -0.000 |
| squared |  |  |  | (0.000) | (0.000) |
| Annualised other expenditure |  |  |  | -0.001 | -0.001 |
|  |  |  |  | (0.004) | (0.004) |
| Annualised other expenditure |  |  |  | 0.000 | 0.000 |
| squared |  |  |  | (0.000) | (0.000) |
| Household durables |  |  |  | -0.000 | -0.000 |
|  |  |  |  | (0.001) | (0.001) |
| continues |  |  |  |  |  |
| House ownership, none |  |  |  | . | . |
| Partially own |  |  |  | -0.999** | -0.941** |
|  |  |  |  | (0.349) | (0.341) |
| Totally own |  |  |  | -0.359* | -0.296 |
|  |  |  |  | (0.174) | (0.171) |
| Length of migration |  |  |  |  | -0.034* |
|  |  |  |  |  | (0.016) |
| Social activity participation, none |  |  |  |  | . |
| One type |  |  |  |  | -0.112 |
|  |  |  |  |  | (0.116) |
| Two types |  |  |  |  | -0.069 |
|  |  |  |  |  | (0.162) |
| Three types |  |  |  |  | -0.083 |
|  |  |  |  |  | (0.604) |
| Health status, excellent as base |  |  |  |  | . |
| Good |  |  |  |  | 0.048 |
|  |  |  |  |  | (0.253) |
| Fair |  |  |  |  | -0.030 |
|  |  |  |  |  | (0.222) |
| Poor |  |  |  |  | 0.603** |
|  |  |  |  |  | (0.228) |
| Very Poor |  |  |  |  | -0.108 |
|  |  |  |  |  | (0.375) |
| Cons | -1.422*** | -1.404*** | -3.195*** | -2.941** | -3.278*** |
|  | (0.070) | (0.172) | (0.886) | (0.936) | (0.965) |
| N | 2963 | 2963 | 2963 | 2963 | 2963 |
| pseudo R-sq | 0.0370 | 0.0370 | 0.0449 | 0.0609 | 0.0766 |

Robust Standard errors in parentheses * p<0.05 **p<0.01 *** p<0.001

Coefficients are in terms of log odds

S4 Decision to Stop Smoking Logistic Regressions (Urban base)

|  | 1 | 2 | 3 | 4 | 5 |
| --- | --- | --- | --- | --- | --- |
| Model specifications | +Age | +Sex | +Early life | +Socioeconomic | Full |
| Urban non-migrants | . | . | . | . | . |
| Urban-to-urban | -0.970* | -0.969* | -1.077* | -1.018* | -1.159* |
|  | (0.437) | (0.436) | (0.458) | (0.447) | (0.569) |
| Urban return migrants | -0.131 | -0.119 | -0.087 | -0.112 | -0.118 |
|  | (0.219) | (0.222) | (0.219) | (0.231) | (0.266) |
| Centered-Age | 0.048** | 0.047** | 0.053*** | 0.041* | 0.042* |
|  | (0.016) | (0.016) | (0.016) | (0.018) | (0.018) |
| Centered-Age squared | 0.001 | 0.001 | 0.001 | 0.001 | 0.001 |
|  | (0.001) | (0.001) | (0.001) | (0.001) | (0.001) |
| Female as base |  | . | . | . | . |
| Male |  | -0.119 | -0.393 | -0.266 | -0.191 |
|  |  | (0.396) | (0.416) | (0.441) | (0.459) |
| Married with spouse present |  |  | . | . | . |
| Married not living with spouse |  |  | -0.339 | -0.240 | -0.166 |
| temporarily |  |  | (0.549) | (0.562) | (0.537) |
| Separated, divorced, widowed and |  |  | -1.067* | -0.914 | -0.848 |
| never married |  |  | (0.461) | (0.470) | (0.479) |
| knee height |  |  | -0.017 | -0.016 | -0.004 |
|  |  |  | (0.028) | (0.027) | (0.030) |
| No formal education |  |  | . | . | . |
| Primary |  |  | 0.583 | 0.491 | 0.472 |
|  |  |  | (0.488) | (0.476) | (0.480) |
| Secondary |  |  | 0.641 | 0.532 | 0.550 |
|  |  |  | (0.511) | (0.488) | (0.483) |
| Tertiary |  |  | 0.910 | 0.665 | 0.643 |
|  |  |  | (0.567) | (0.551) | (0.559) |
| First job as farmers base |  |  | . | . | . |
| First job in the Government |  |  | -0.152 | -0.192 | 0.044 |
|  |  |  | (0.478) | (0.477) | (0.483) |
| First job in Institutions |  |  | -0.180 | -0.254 | -0.173 |
|  |  |  | (0.376) | (0.405) | (0.432) |
| First job in State firms |  |  | 0.374 | 0.334 | 0.297 |
|  |  |  | (0.266) | (0.281) | (0.282) |
| First job in Individual Firms |  |  | -0.418 | -0.493 | -0.434 |
|  |  |  | (0.548) | (0.562) | (0.542) |
| First job in other jobs |  |  | 0.234 | 0.176 | 0.267 |
|  |  |  | (0.478) | (0.462) | (0.456) |
| Current job status, agricultural work |  |  |  | . | . |
| Wage work |  |  |  | -0.089 | 0.074 |
|  |  |  |  | (0.328) | (0.334) |
| Retired and receive any pension |  |  |  | 0.299 | 0.249 |
|  |  |  |  | (0.339) | (0.350) |
| Retired and receive no pension |  |  |  | 0.132 | 0.053 |
|  |  |  |  | (0.470) | (0.501) |
| Not working |  |  |  | -0.772 | -0.826* |
|  |  |  |  | (0.422) | (0.416) |
| Annualised food expenditure |  |  |  | 0.043* | 0.043* |
|  |  |  |  | (0.018) | (0.018) |
| Annualised food expenditure |  |  |  | -0.000 | -0.000 |
| squared |  |  |  | (0.000) | (0.000) |
| Annualised other expenditure |  |  |  | 0.007 | 0.006 |
|  |  |  |  | (0.005) | (0.005) |
| Annualised other expenditure |  |  |  | -0.000 | -0.000 |
| squared |  |  |  | (0.000) | (0.000) |
| Household durables |  |  |  | -0.008 | -0.008 |
|  |  |  |  | (0.006) | (0.005) |
| continues |  |  |  |  |  |
| House ownership, none |  |  |  | . | . |
| Partially own |  |  |  | -1.165 | -1.353 |
|  |  |  |  | (0.678) | (0.787) |
| Totally own |  |  |  | -0.036 | -0.050 |
|  |  |  |  | (0.307) | (0.311) |
| Length of migration |  |  |  |  | 0.010 |
|  |  |  |  |  | (0.015) |
| Social activity participation, none |  |  |  |  | . |
| One type |  |  |  |  | 0.181 |
|  |  |  |  |  | (0.245) |
| Two types |  |  |  |  | 0.379 |
|  |  |  |  |  | (0.300) |
| Three types |  |  |  |  | -0.011 |
|  |  |  |  |  | (0.479) |
| Health status, excellent as base |  |  |  |  | . |
| Good |  |  |  |  | 0.259 |
|  |  |  |  |  | (0.547) |
| Fair |  |  |  |  | 0.789 |
|  |  |  |  |  | (0.508) |
| Poor |  |  |  |  | 1.368* |
|  |  |  |  |  | (0.551) |
| Very Poor |  |  |  |  | 2.112** |
|  |  |  |  |  | (0.783) |
| Cons | -1.114*** | -1.008** | -0.598 | -0.962 | -2.567 |
|  | (0.158) | (0.390) | (1.386) | (1.381) | (1.653) |
| N | 676 | 676 | 676 | 676 | 676 |
| pseudo R-sq | 0.0421 | 0.0423 | 0.0671 | 0.0965 | 0.1250 |

Robust Standard errors in parentheses * p<0.05 **p<0.01 *** p<0.001

Coefficients are in terms of log odds

S5 Decision to Smoke Logistic Regressions (rural non-migrants vs urban non-migrants)

|  | 1 | 2 | 3 | 4 | 5 |
| --- | --- | --- | --- | --- | --- |
| Model specifications | +Age | +Sex | +Early-life | +Socioeconomic | Full |
| Rural non-migrants | . | . | . | . | . |
| Urban non-migrants | 0.698*** | 0.597*** | 0.672** | 0.701* | 0.697* |
|  | (0.601-0.809) | (0.486-0.733) | (0.517-0.874) | (0.528-0.931) | (0.527-0.923) |
| N | 7100 | 7100 | 7100 | 7100 | 7100 |
| pseudo R-sq | 0.0076 | 0.3822 | 0.3870 | 0.3890 | 0.3915 |

odds ratios in the table * p<0.05 **p<0.01 *** p<0.001

95% confidence intervals in parentheses, calculated based on robust standard errors

S6 Decision to Stop Smoking Logistic Regressions (rural non-migrants vs urban non-migrants)

|  | 1 | 2 | 3 | 4 | 5 |
| --- | --- | --- | --- | --- | --- |
| Model specifications | +Age | +Sex | +Early life | +Socioeconomic | Full |
| Rural non-migrants | . | . | . | . | . |
| Urban non-migrants | 1.454* | 1.452* | 1.293 | 1.052 | 1.101 |
|  | (1.092-1.938) | (1.089-1.936) | (0.930-1.799) | (0.729-1.519) | (0.766-1.583) |
| N | 2774 | 2774 | 2774 | 2774 | 2774 |
| pseudo R-sq | 0.0130 | 0.0135 | 0.0220 | 0.0327 | 0.0506 |

odds ratios in the table * p<0.05 **p<0.01 *** p<0.001

95% confidence intervals in parentheses, calculated based on robust standard errors
